# Biomarkers allow detection of nutrient limitations and respective supplementation for elimination in *Pichia pastoris* fed-batch cultures

**DOI:** 10.1186/s12934-017-0730-9

**Published:** 2017-07-11

**Authors:** Jonas Burgard, Minoska Valli, Alexandra B. Graf, Brigitte Gasser, Diethard Mattanovich

**Affiliations:** 1 Austrian Centre of Industrial Biotechnology, Vienna, Austria; 2 Department of Biotechnology, BOKU-University of Natural Resources and Life Sciences, Muthgasse 18, 1190 Vienna, Austria; 3 School of Bioengineering, University of Applied Sciences FH Campus Vienna, Vienna, Austria

**Keywords:** *Pichia pastoris*, Yeast, Transcriptional regulation, Fed batch cultivation, Transcriptomics, Media optimization, Starvation, Nutrient limitation, Marker genes

## Abstract

**Background:**

Industrial processes for recombinant protein production challenge production hosts, such as the yeast *Pichia pastoris*, on multiple levels. During a common *P. pastoris* fed-batch process, cells experience strong adaptations to different metabolic states or suffer from environmental stresses due to high cell density cultivation. Additionally, recombinant protein production and nutrient limitations are challenging in these processes.

**Results:**

*Pichia pastoris* producing porcine carboxypeptidase B (CpB) was cultivated in glucose or methanol-limited fed-batch mode, and the cellular response was analyzed using microarrays. Thereby, strong transcriptional regulations in transport-, regulatory- and metabolic processes connected to sulfur, phosphorus and nitrogen metabolism became obvious. The induction of these genes was observed in both glucose- and methanol- limited fed batch cultivations, but were stronger in the latter condition. As the transcriptional pattern was indicative for nutrient limitations, we performed fed-batch cultivations where we added the respective nutrients and compared them to non-supplemented cultures regarding cell growth, productivity and expression levels of selected biomarker genes. In the non-supplemented reference cultures we observed a strong increase in transcript levels of up to 89-fold for phosphorus limitation marker genes in the late fed-batch phase. Transcript levels of sulfur limitation marker genes were up to 35-fold increased. By addition of (NH_4_)_2_SO_4_ or (NH_4_)_2_HPO_4_, respectively, we were able to suppress the transcriptional response of the marker genes to levels initially observed at the start of the fed batch. Additionally, supplementation had also a positive impact on biomass generation and recombinant protein production. Supplementation with (NH_4_)_2_SO_4_ led to 5% increase in biomass and 52% higher CpB activity in the supernatant, compared to the non-supplemented reference cultivations. In (NH_4_)_2_HPO_4_ supplemented cultures 9% higher biomass concentrations and 60% more CpB activity were reached.

**Conclusions:**

Transcriptional analysis of *P. pastoris* fed-batch cultivations led to the identification of nutrient limitations in the later phases, and respective biomarker genes for indication of limitations. Supplementation of the cultivation media with those nutrients eliminated the limitations on the transcriptional level, and was also shown to enhance productivity of a recombinant protein. The biomarker genes are versatily applicable to media and process optimization approaches, where tailor-made solutions are envisioned.

**Electronic supplementary material:**

The online version of this article (doi:10.1186/s12934-017-0730-9) contains supplementary material, which is available to authorized users.

## Background

Production of heterologous proteins in yeast becomes more and more important in the biotechnological sector for research use, therapeutics or sustainable energy sources [1, 2]. This development has led to the constant demand for improvement of hosts and processes for recombinant protein production (RPP). Common approaches for improvement of RPP are mostly based on strain engineering [3], process optimization [4] including adaptation of culture conditions [5–8], feed strategies [9], or media optimization [10, 11]. As production processes become more and more specific due to special needs of production hosts [11] and the recombinant product, media need to be developed for a specific process and are often tailor-made [12]. In general, cultivation media should be chemically defined, however, high level protein synthesis, influencing cell physiology, might make supplementation with e.g. amino acids necessary [13]. For bioreactor cultivations of the yeast production host *Pichia pastoris* (*Komagataella* spp.) different cultivation media have been developed. The original basal salt medium (BSM) [14] is a standard medium but comprises an unbalanced composition, tends to salt precipitation [15], and has a high ionic strength [12]. Adapted or alternative media have been tested by Zhang et al. [15], Kobayashi et al. [5], Zhao et al. [16], d'Anjou [17], Stratton et al. [18], or Maurer et al. [19] and also optimization approaches for *P. pastoris* biomass production have been published [20]. In this context challenges for media composition have been described. The modified basal salt medium (BSM) from Zhang et al. [15] e.g. might be problematic due to insufficient nutrient supply during growth upon precipitation of phosphorus compounds. Furthermore it has been reported that osmotic stress can lead to induction of the unfolded protein response [21] and high salinity may even inhibit growth or protein synthesis [22]. Such high salt concentrations can lead to loss of internal cellular pressure, effects on membrane transporter activity, ion homeostasis and internal pH-equilibrium finally leading to protein misfolding and reactive oxygen species (ROS) generation [22]. Another problem might occur during downstream processing as high salt containing supernatants may need to be diluted to adapt conductivity for ion exchange chromatography [4].

Commonly performed industrial processes for production of recombinant proteins using a production host in a carbon-limited fed-batch can lead to starvation conditions at high biomass concentrations [11]. In this context it was already reported that downregulation of the protein synthesis machinery e.g. ribosome biogenesis [23, 24] and the secretion apparatus [25] occurred in starvation conditions in yeast processes, both having direct impact on product yield. The development of a cultivation medium can be achieved by e.g. determination of the elemental composition [26, 27] or an optimization by design of experiment (DoE)-approaches [28] which is more efficient than the traditional linear optimization process [29]. In such a setup, different supplements and concentrations can systematically be tested to find the optimum. The time required to customize a culture medium for a particular cell line depends on the goals [30].

Such studies indicate the need for tailor-made cultivation media for industrially relevant production strains and suitable methods to detect nutrient limitations. Studies on cellular adaptations upon starvation conditions have been already performed for model organisms e.g. *Saccharomyces cerevisiae* [31], but no data are available for cultivation of *P. pastoris* strains in a production process for recombinant proteins yet. Omics analyses so far have been used to understand the response of *P. pastoris* to different carbon sources [32, 33], growth rates [34] and environmental conditions [35]. Furthermore it helped to understand the cellular reactions to recombinant protein production [36] and the results were used for strain engineering. Only the findings of Baumann et al. [8] were used for process design. As a complete characterization of the transcriptional regulatory patterns of a fed-batch process has not been performed so far, we set out to determine the gene expression levels of a strain cultivated either in a glucose or methanol-limited fed-batch, and use the regulatory insights to improve the production process.

## Results

### Methanol- and glucose-limited fed-batch cultivation of recombinant *P. pastoris* with equal biomass generation to allow comparable processes for transcriptomics analysis

**Fig. 1:**
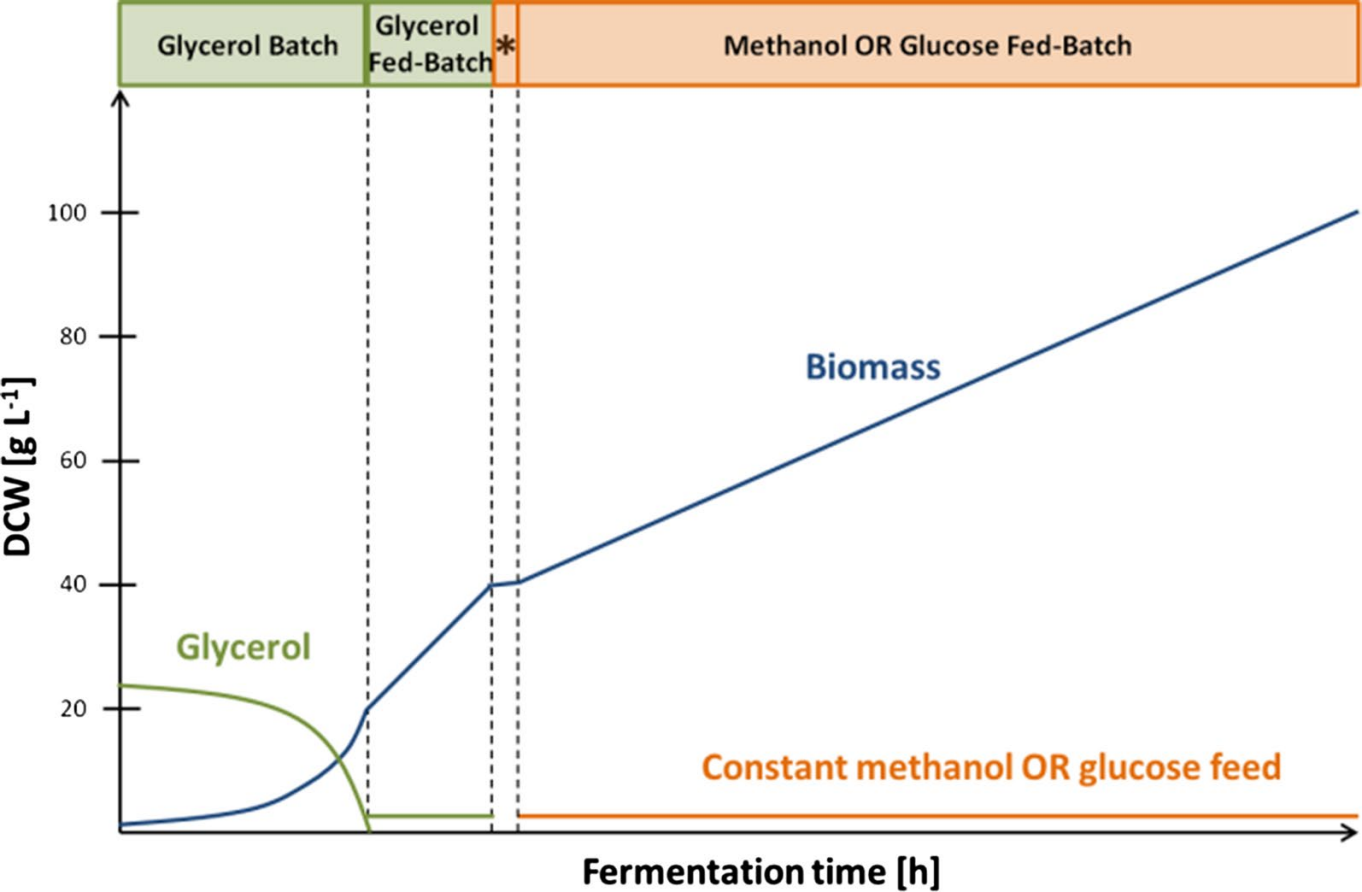
Schematic representation of the fed-batch cultivations for transcriptional analysis of *P. pastoris*. The process comprised a glycerol batch phase, followed by a limiting glycerol fed-batch for 5 h to double the biomass. Then methanol-grown cultures received a methanol pulse, while glucose-grown cultures were slowly fed with glucose to accumulate the same amount of biomass during this period (*Asterisk*). Afterwards a constant methanol or glucose feed was applied

*Pichia pastoris*, producing porcine pro-carboxypeptidase B (CpB) under the control of the P_GAP_-promoter was cultivated in standard methanol and glucose fed-batch processes (Fig. 1). In order to ensure comparability one strain that constitutively produces the recombinant product was chosen for both the glucose- as well as the methanol-based cultivations, and the processes were designed for equal biomass generation and feed volumes. The processes comprised a glycerol batch phase (IAM medium), followed by a limiting glycerol feed (GLY01 medium), subsequently the cultures were either pulsed with methanol for induction of the methanol metabolism or a low constant glucose feed of GLU04 medium was started. After consumption of the pulse, the methanol-grown cultures received a constant methanol feed (MET01 medium), while the glucose-grown cultures received a constant glucose feed for 67 h. These media, described in detail in the “Methods” section, were successfully used for several recombinant protein production processes before. Both processes were performed in triplicates. Samples were taken in regular intervals and characterized for growth and CpB production. Furthermore, transcriptome analyses were performed to study the cellular response over the course of the production processes.

**Fig. 2:**
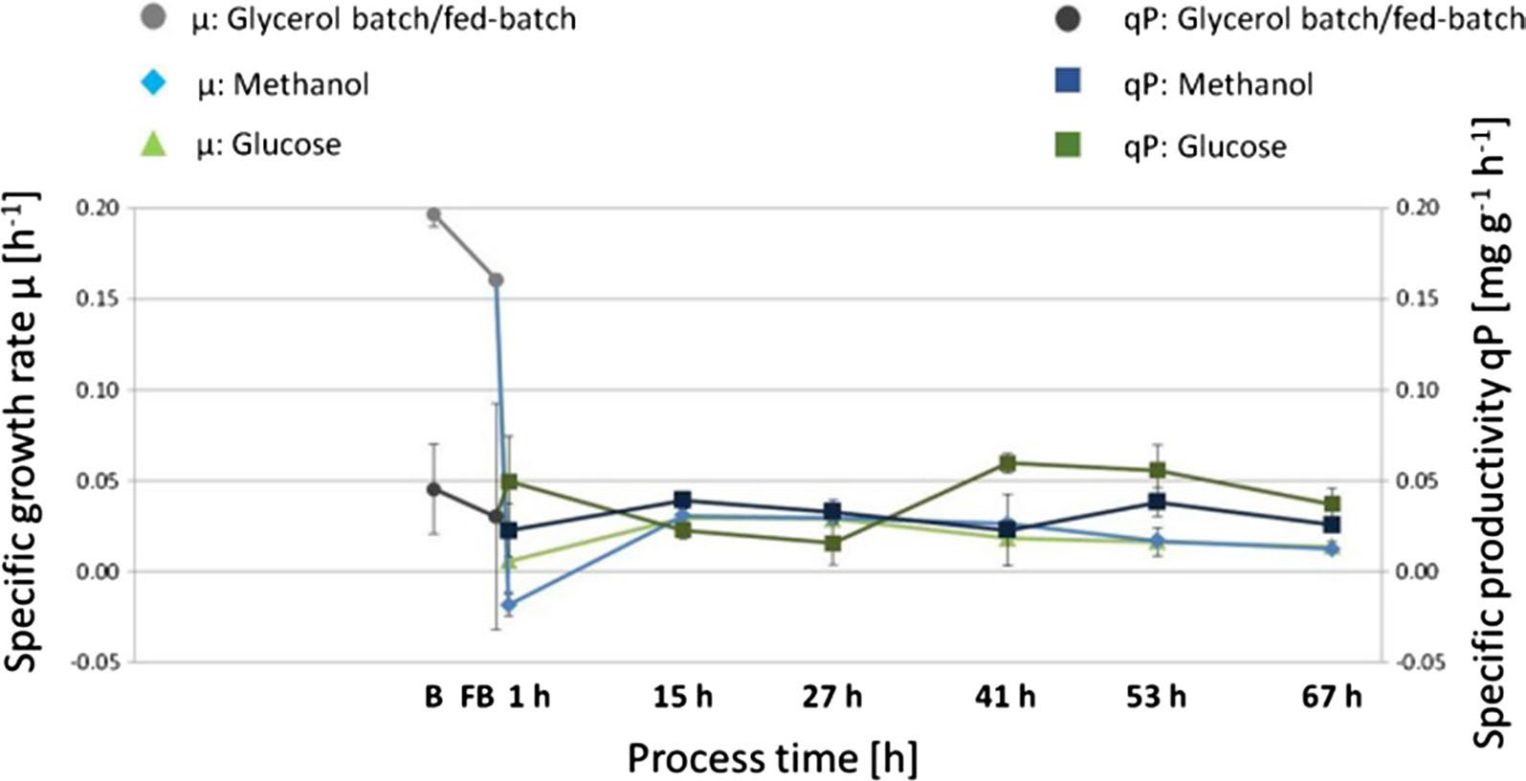
Specific growth rate (h^−1^) and specific productivity (mg g^−1^ h^−1^) of CpB producing *P. pastoris* in methanol or glucose-limited fed batch cultivations (*B* Glycerol batch; *FB* Glycerol fed-batch; 1–67 h glucose- or methanol fed-batch; B and FB: 6 biological replicates; glucose or methanol FB: 3 biological replicates). *Error bars* indicate standard deviation

The biomass (dry cell weight; DCW) reached 21 (±1) g L^−1^ after the glycerol batch and 44 (±2) g L^−1^ after glycerol fed-batch. After 67 h, methanol-grown cultures reached 103 (±5) g L^−1^ DCW and glucose-grown cultures 103 (±3) g L^−1^ DCW. The culture viability remained high throughout the glucose and methanol-based processes (>96%). During the process, the specific growth rate decreased from 0.2 to 0.01 h^−1^ upon the increasing carbon limitation (Fig. 2). Specific productivity differed during the adaptation phase for methanol metabolism and the very low glucose feed. For the methanol-limited fed batch it stayed stable at about 0.03 mg g^−1^ h^−1^, whereas for the glucose-limited processes it was higher in the second half of the feed phase, compared to the first phase.

### Strong transcriptional response upon change of carbon source and increase in gene regulation in high cell density cultures is occurring in *P. pastori*s fed batch cultivations

**Fig. 3:**
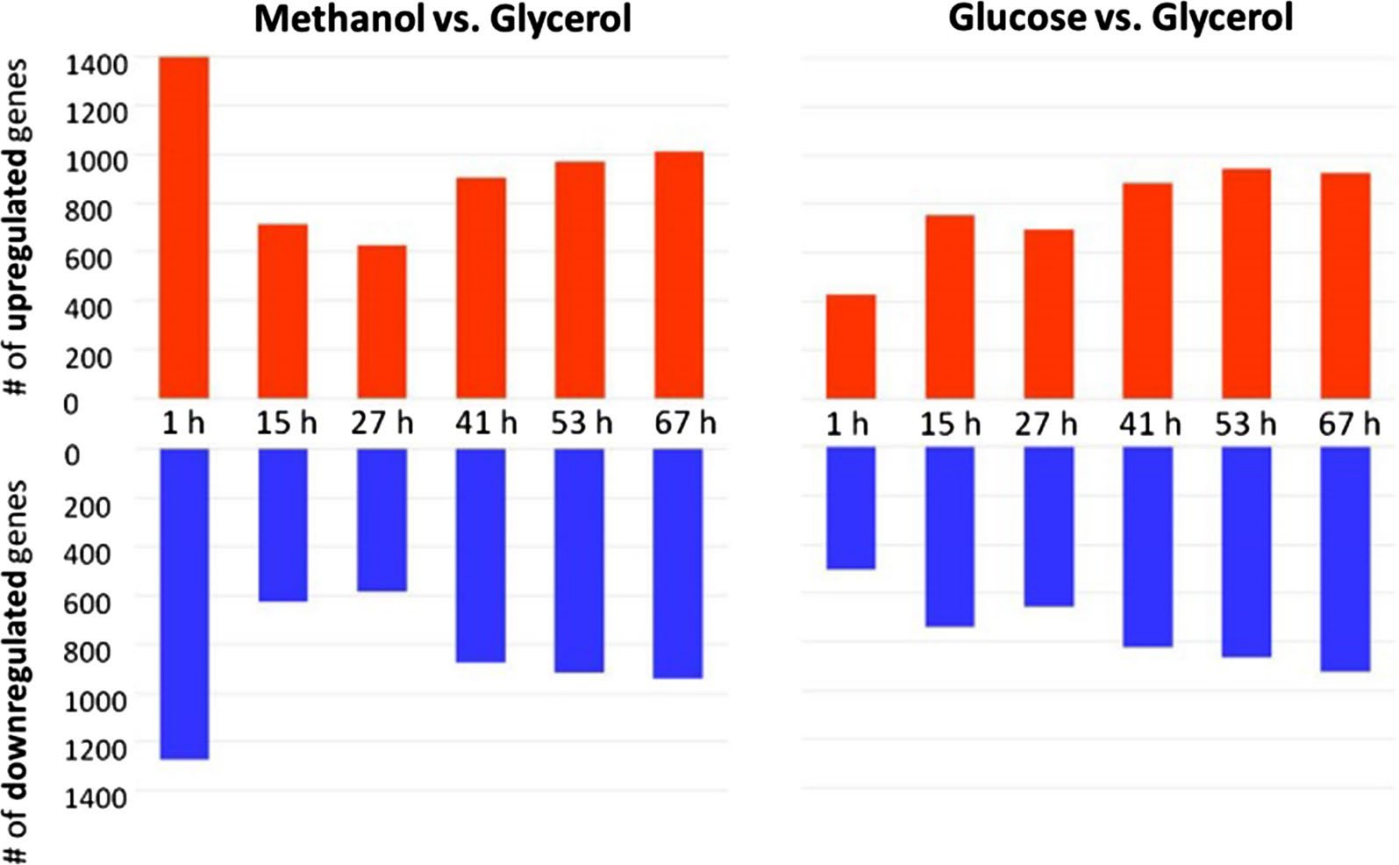
Number of differentially regulated genes (*up* or *down*) in the methanol or glucose fed-batch compared to the glycerol fed-batch. The x-axis shows the time course of methanol and glucose fed-batch in h

*Pichia pastoris* specific microarray analyses were performed of samples from the end of the glycerol batch, the end of glycerol fed-batch and from methanol or glucose fed-batches (1, 15, 27, 41, 53 and 67 h after starting the constant feed) and expression patterns were investigated. Differentially regulated genes (log_2_FC ≤ −0.58 OR ≥ 0.58; AND adjusted P value <0.05) were determined taking the end of the glycerol fed-batch phase, as last common condition, as reference. The numbers of differentially regulated genes are shown in Fig. 3. In the methanol fed-batch we observed a strong induction of gene regulation (significant up- and downregulation) 1 h after starting the methanol feed (50%) correlating to restructuring of metabolism towards methanol utilization. This is in contrast to the early phase of the glucose fed-batch where we observed a lower degree of gene regulation (17%). After the early adaptation phase both processes show the same trend for increased gene regulation from the middle (25%) to the end of the fed-batch processes (~35%). The proportion of up- and downregulated genes is very similar and follows the same trend during the fed-batch process on both carbon sources. A probe for the CpB gene was also included on the microarrays and expression remained constantly high throughout the cultivation, both on methanol and glucose as carbon source (Additional file 1: Figure S1).

**Fig. 4:**
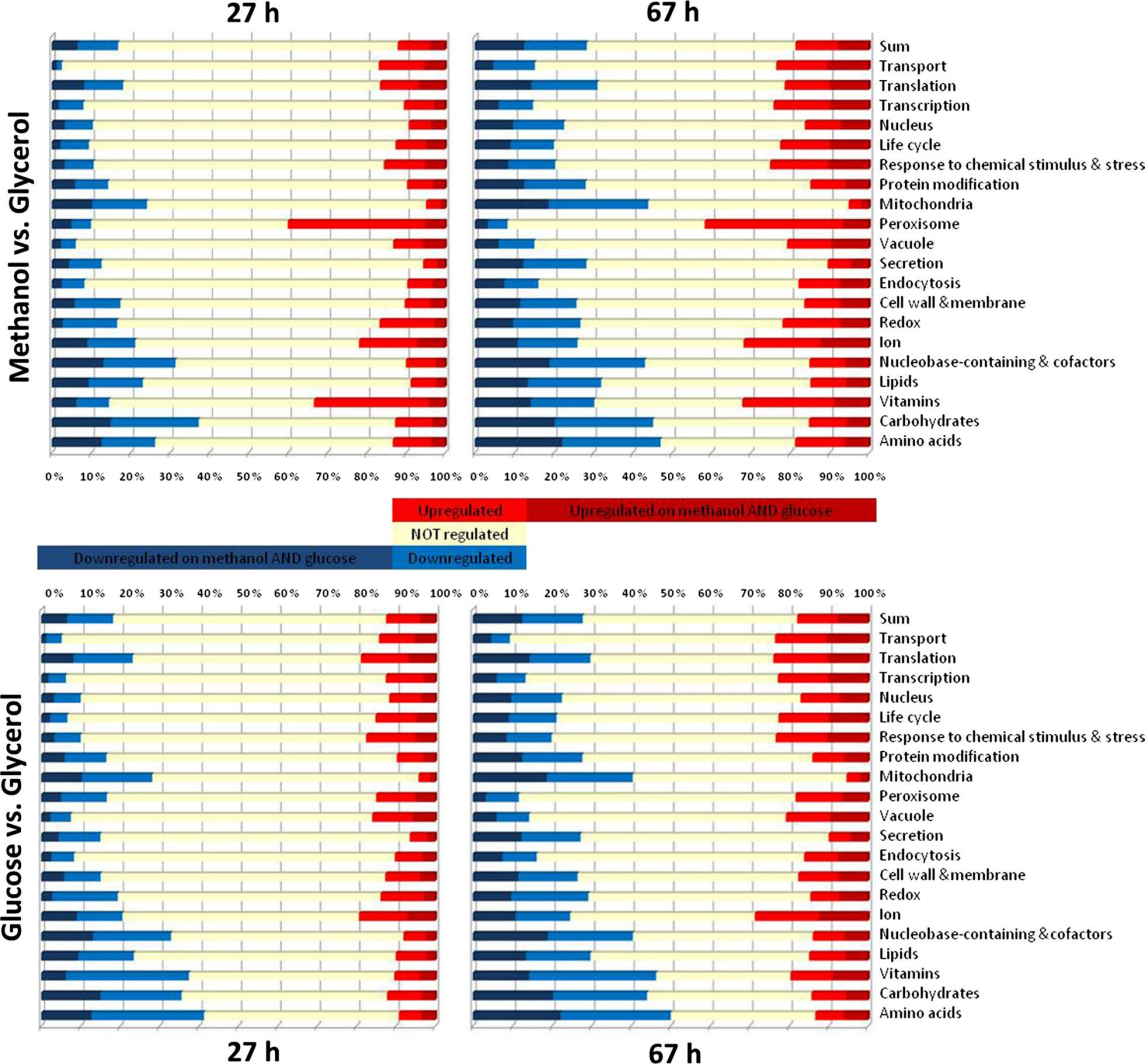
Functional categories (GO slim terms) with significant transcriptional regulation in methanol- or glucose-fed-batch (27 or 67 h) in comparison to the glycerol fed-batch. Significant regulation: log_2_FC ≤ −0.58 OR ≥ 0.58 AND adjusted P value <0.05

**Fig. 5:**
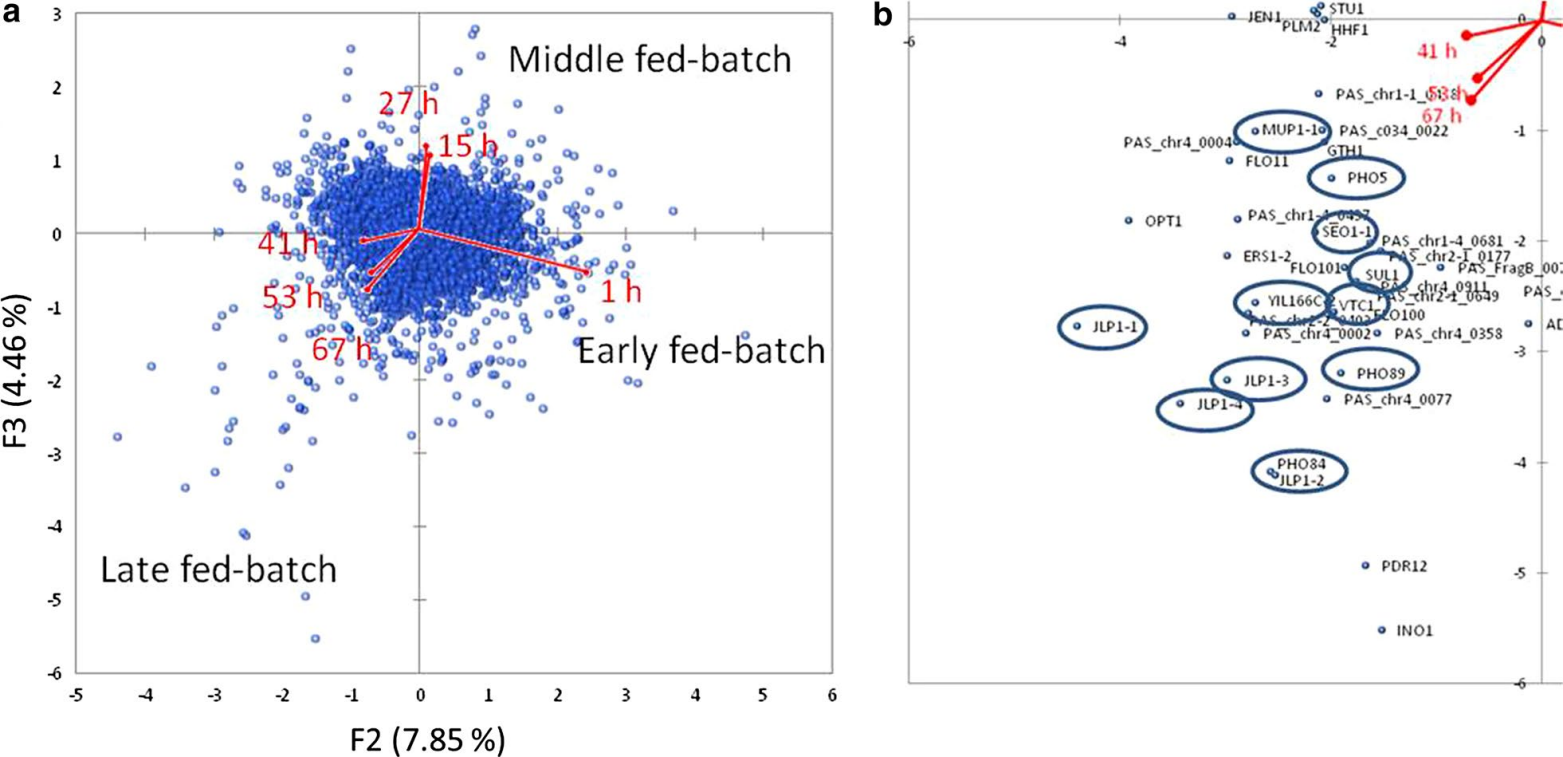
Principal component analysis (PCA) bi-plots of the methanol fed-batch (1, 15, 27, 41, 53 and 67 h after starting the methanol fed-batch) referred to the glycerol fed-batch (log_2_ fold change). **a** *Vectors* indicate variables (sampling time points) and *blue data points* indicate individuals (genes). **b** Excerpt from quadrant 4 with genes that were strongly regulated in the later fed-batch phase (reduced for individuals with variance below 2%). Genes involved in metabolism of nitrogen, phosphorus and sulfur are highlighted by *circles*

For all comparisons, GO term analysis was performed to identify overrepresented functional categories (Fig. 4), while principal component analysis (PCA) revealed similarities between the different analysed time points (Fig. 5).

For generation of a compact overview of the transcriptional response to the carbon limited fed-batch conditions at high cell densities (up to 100 g L^−1^ DCW), we grouped significantly regulated genes in the middle (27 h) and the end of the methanol and glucose fed-batch (67 h) for their cellular functions using GO-slim terms (http://www.yeastgenome.org/help/analyze/go-slim-mapper) (Fig. 4). This excerpt of the fed-batch process is shown exemplarily as the methanol and glucose fed-batch can be divided by their transcriptional response in an early (1 h), middle (15, 27 h) and late fed-batch phase (41, 53, 67 h) using PCA; (Fig. 5 and Additional file 1: Figures S4–S7).

Analyzing the time course of the methanol or glucose fed-batch (27 and 67 h) those graphs show an increasing number of downregulated genes associated with translation, correlating with the decreasing carbon source availability and specific growth rate. Furthermore an increasing regulation in cell cycle genes, the downregulation of energy metabolism (mitochondria) and of pathways associated with nucleobases and cofactors, lipids, carbohydrates and amino acids was observed in both conditions over the time course. The strongest differences between methanol and glucose grown cells were in the upregulation of peroxisomal genes and vitamins (see Russmayer et al. [33]) which were strongly upregulated during the methanol fed-batch. Interestingly also many transport pathways (endocytosis, ions) and pathways, including metabolic recycling for providing alternative substrates (e.g. amino acids) by the vacuole as replacement for preferred substrates were strongly transcriptionally regulated. This number even increased from the middle (27 h) to the later fed-batch (67 h) and was more pronounced in the methanol fed-batch, compared to the glucose fed-batch.

### Transcriptome data reveal limitations of the macroelements sulfur, phosphorus and nitrogen

For further investigation of the changes in gene regulation being representative for the late phases of the fed–batch processes (41, 53, and 67 h), we inspected quadrant 4 of the PCA plot (Fig. 5b). In this analysis we identified many genes associated with sulfur, phosphorus and nitrogen metabolism indicating for nutrient limitations. Genes involved in sulfur metabolism, include *JLP1*-*1*, *JLP1*-*2*, *JLP1*-*3*, *SUL1*, *YIL166C2*, and *MUP1*-*1*. Phosphorus-associated genes were *PHO5*, *PHO84*, *PHO89*, and *VTC1*. Nitrogen-associated genes were e.g. *DUR3*-*1*, *SEO1*, and *YBR139W*.

**Fig. 6:**
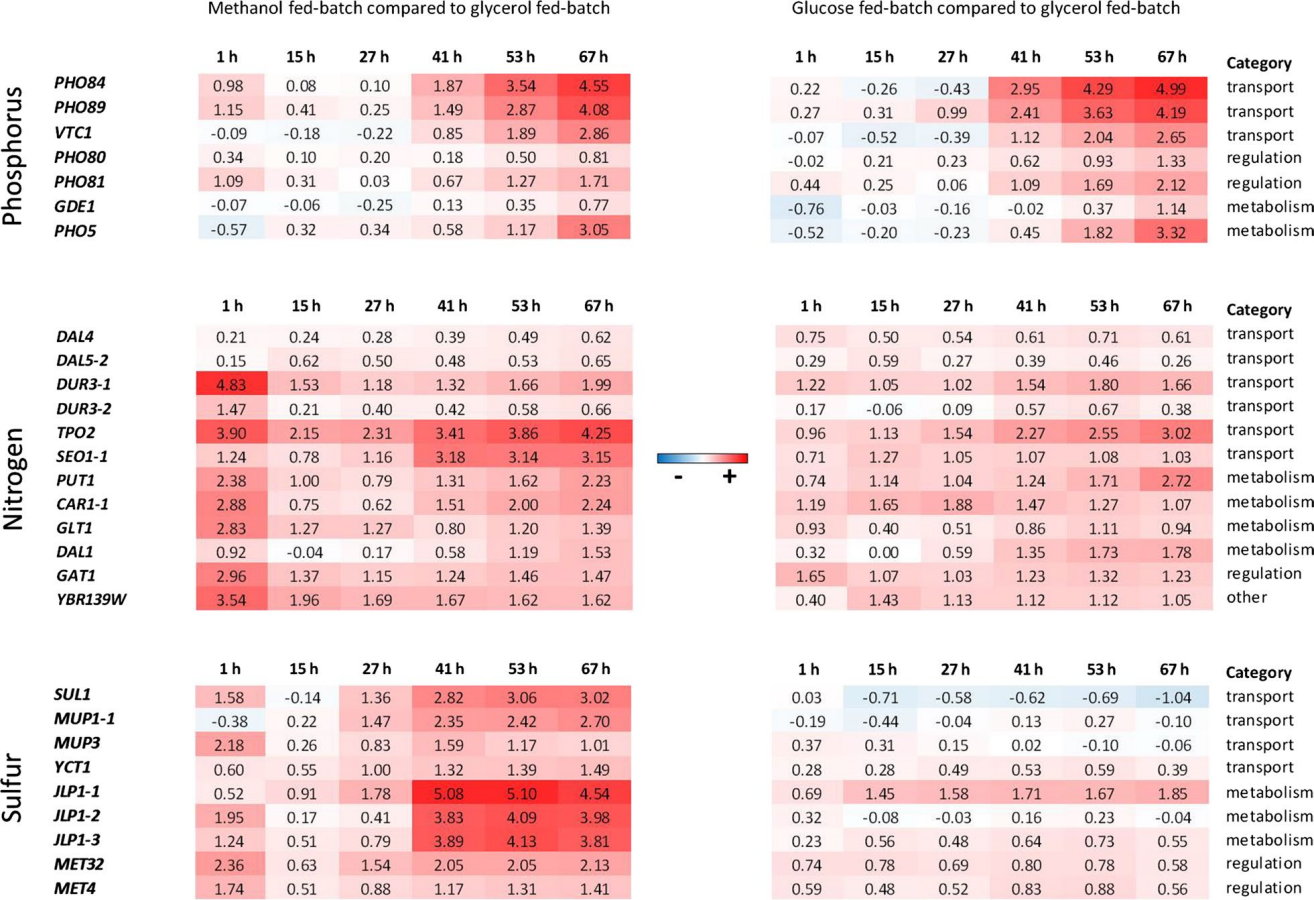
Heat maps (*red* upregulated; *white* not regulated; *blue* downregulated) depicting the regulatory trends of potential marker genes for nutrient limitation during the methanol or glucose fed-batch process. Genes involved in transport, metabolism and regulation of phosphorus, nitrogen, and sulfur are shown. Log_2_FC are calculated compared to transcript levels of glycerol FB. *More intense coloring* indicates stronger regulation

For further investigation of the transcriptomics data we focused on those genes and made a selection of genes involved in metabolism, regulatory and transport processes. Transcriptomics data of all genes associated with limitations of these nutrients are shown in log_2_ fold changes in the heat maps in Fig. 6 (description of biological function in Additional file 1: Table S2).

Transcriptional regulation of these genes, compared to the glycerol fed-batch, mostly followed a similar trend in the methanol fed-batch since expression levels were strongly induced upon the change of the carbon source (1 h), then they decreased to a not or only slightly differentially regulated state, and later on strongly increased from the middle to the end of the fed-batch (41–67 h). The highest changes were observed for the phosphate transporter *PHO84* with a log_2_FC of 4.55 corresponding to a 23-fold upregulation. Summarizing the functional distribution, mainly gene products involved in nutrient uptake, metabolism of alternative/non-preferred sources of the respective nutrients and correlating upregulation of the respective pathways were found. As already described for the overall transcriptional regulation in the glucose fed-batch, there is just a weak induction of gene regulation upon the change of carbon source. During the late fed-batch, increasing upregulation of potential marker genes for sulfur-limitation was less pronounced whereas induction of the selected phosphorus-associated genes was comparable to the observed level of upregulation during the methanol fed-batch. And also nitrogen associated genes followed the same trend, albeit having a weaker induction.

Based on PCA analyses and the pronounced regulatory trend of gene regulation displayed in the heat maps, nutrient limitations can be assumed. Therefore, we analyzed and compared the media composition of our medium with different published cultivation media. Indeed, this comparison revealed strong differences in sulfur content (2–112 mM) and phosphorus levels (21–395 mM) in the batch medium [5, 16, 18, 19]. Such differences, resulting in a high salt content of the medium, have already been shown to be problematic for cellular growth. Besides sulfur and phosphorus, we additionally analyzed nitrogen content. As ammonium serves as pH control and nitrogen source in *P. pastoris* bioreactor cultivations the supply is herewith strongly strain- and process-dependent and not directly comparable.

### Medium supplementation leads to improved biomass generation and influences CpB-production

Based on the transcriptomics data and comparison of media recipes we decided to supplement *P. pastoris* methanol-grown cultures with (NH_4_)_2_HPO_4_ or (NH_4_)_2_SO_4_. The supplements were added as pulses during the cultivation due to limited solubility in the methanol-feed medium. The added amounts represented the difference between the used medium and the BSM medium or the doubled amount (2×), however, contrary to the BSM medium the supplements were not included into the batch medium but added as pulses during the fed batch phase. To evaluate the effects of the supplementations, we selected four potential marker genes per compound addressing different aspects of molecular function and exhibiting strong changes in transcript levels during the fed-batch process. Those genes were *PHO5, PHO81, PHO84* and *VTC1* for phosphorus limitation, *JLP1*-*1*, *MET32*, *SUL1* and *MUP1*-*1* for sulfur limitation, and *DUR3*-*1*, *TPO2*, *PUT1*, *DAL1* for nitrogen limitation.

**Fig. 7:**
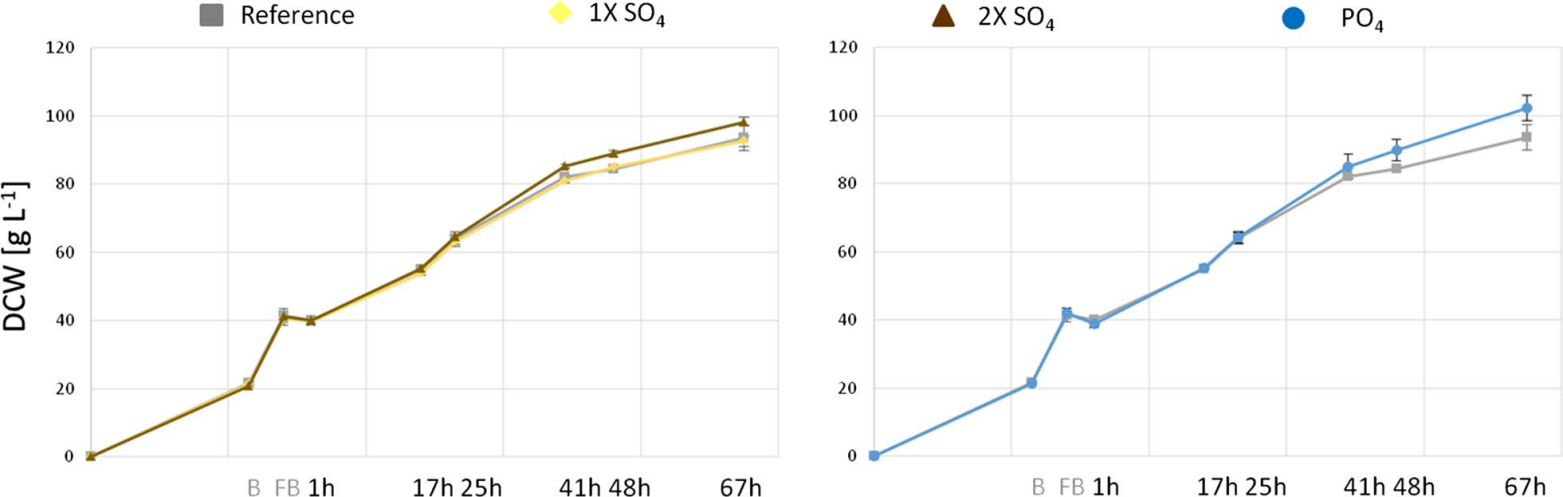
Biomass generation (DCW) in the unsupplemented reference cultures, 1× SO_4_, 2× SO_4_- and 1× PO_4_-supplemented culture. *B* glycerol batch, *FB* glycerol fed-batch; 1–67 h methanol fed-batch. *Error bars* indicate standard deviation of the duplicate fed batch cultivations

To see if the addition of phosphorus, sulfur or nitrogen can diminish the transcriptional upregulation and has an impact on biomass formation and productivity, bioreactor cultivations were performed in duplicates and analyzed for biomass generation (DCW) and production of secreted CpB. In Fig. 7 the biomass development of the reference culture and the supplemented cultures are shown. 1× or 2× SO_4_-supplementation correlates with addition of 23 or 46 mM sulfur and 1× PO_4_-supplementation with 28 mM additional phosphorus. Due to their limited solubility in methanol, supplements were given after each sampling, beginning with the end of the glycerol fed-batch. Biomass generation in biological replicates was reproducible and 2× SO_4_ and PO_4_-supplemented cultures showed an improved growth with 4 g L^−1^ (5%) higher DCW in the 2× SO_4_-supplemented cultures and 8 g L^−1^ (9%) higher DCW for the PO_4_-supplemented culture. 1× SO_4_ supplementation did not lead to improved biomass generation.

**Fig. 8:**
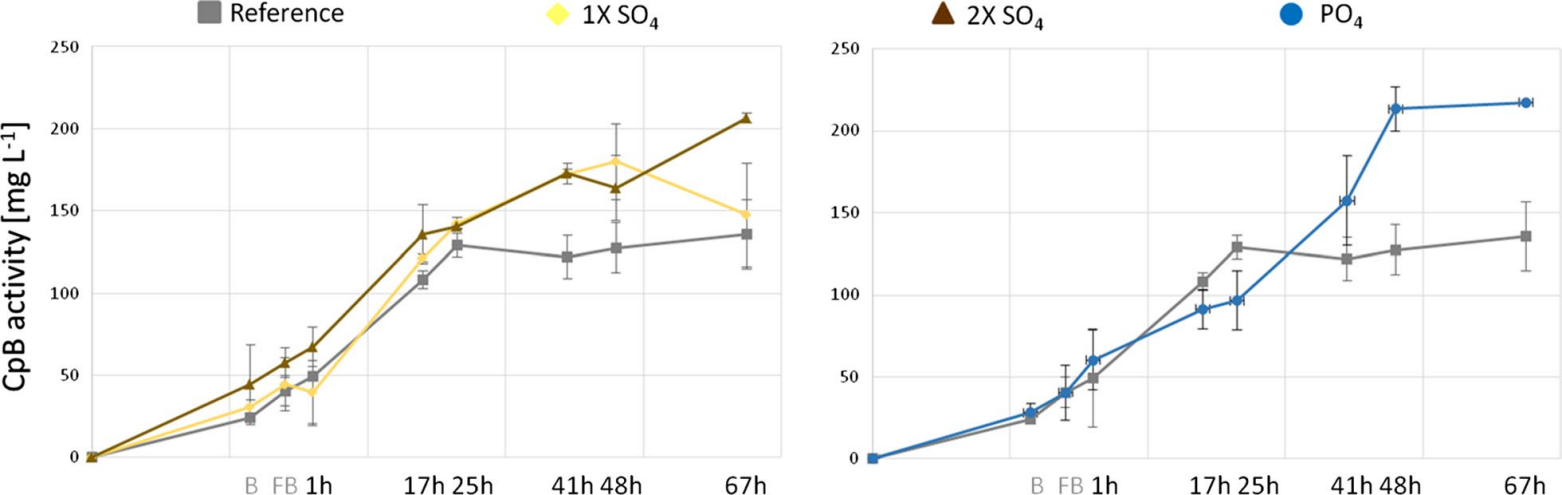
Total CpB-activity in the reference-, 1× SO_4_-, 2× SO_4_- and 1× PO_4_-supplemented fed-batch cultures. (*B* glycerol batch, *FB* glycerol fed-batch; 1–67 h methanol fed-batch). *Error bars* indicate standard deviation of the duplicate cultures

CpB-activity was determined in all culture supernatants (Fig. 8) using an enzymatic assay. As we used the constitutive P_GAP_ promoter CpB levels are expected to increase during the whole process. In the reference cultivations (without any supplement) we observed a plateau in CpB activity during the later fed-batch and a final CpB titer of 136 mg L^−1^on average. In the 1× SO_4_-supplemented cultures product titers rose until 48 h of fed-batch, but then declined until the end of the process to levels slightly higher than the reference culture (148 mg L^−1^; 9% improvement). In contrast, 2× SO_4_-supplemented cultures reached 206 mg L^−1^ CpB (52% improvement) and 1× PO_4_-supplemented cultures even 217 mg L^−1^ CpB (60% improvement). SDS-PAGEs confirmed the trend of the activity values. Viability of the cultures was measured at the end of the cultivations and remained comparably high (>97%) in all cultivations.

### Sulfur and phosphorus supplementation cures upregulation of marker gene transcript levels during the fed batch

**Fig. 9:**
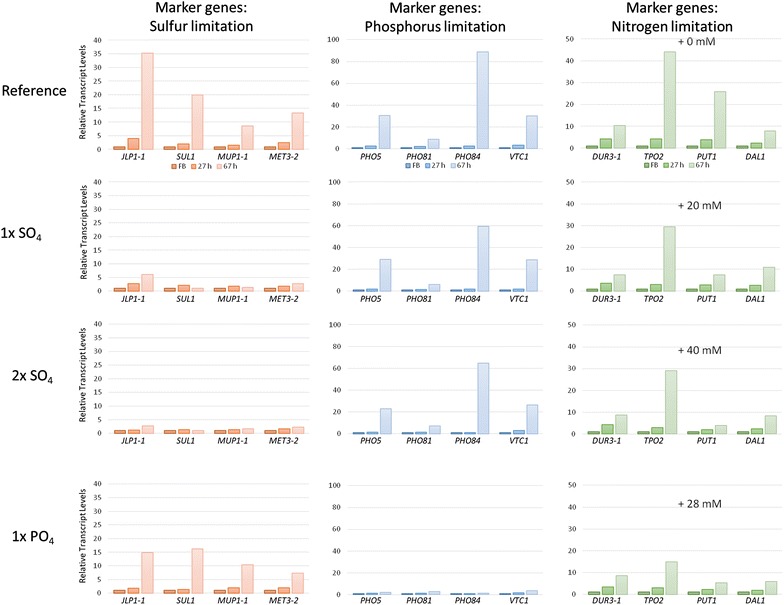
Relative transcript levels of selected marker genes for sulfur-, phosphorus-, and nitrogen limitation in the reference and supplemented cultivations (1× SO4, 2× SO_4_, 1× PO_4_). Transcript levels are normalized to ACT1 housekeeping gene and transcript levels of the middle (27 h) and end of the methanol fed-batch (67 h) are further normalized to the glycerol fed-batch (FB)

Transcript levels of the potential nutrient limitation marker genes were determined by quantitative PCR (qPCR) in the reference culture (no supplement) and the supplemented cultures. Transcript levels at the middle (27 h) and the end (67 h) of the methanol fed-batch are shown as fold changes to the glycerol fed-batch in Fig. 9. In the reference cultures we were able to verify the transcriptional regulatory patterns for the selected marker genes that were observed with microarrays in the previous cultivations. The strongest marker gene for sulfur limitation (*JLP1*-*1*) showed an increase of up to 35-fold from the middle to the end of fed-batch in the reference culture, and the other marker genes were 10- to 20-fold induced. Transcript levels of the strongest phosphorus limitation marker gene (*PHO84*) even increased up to about 89-fold, while *PHO5* and *VTC1* were approximately 30-fold upregulated.

In 1× (NH_4_)_2_SO_4_-supplemented cultures, upregulation of transcript levels of sulfur limitation marker genes was strongly reduced compared to the reference culture. By addition of the double amount (NH_4_)_2_SO_4_ no upregulation was observed, and transcript levels were nearly suppressed to the levels of the glycerol fed-batch. Similarly, in (NH_4_)_2_HPO_4_-supplemented cultures transcript level of phosphorus limitation marker genes were reduced to the glycerol fed-batch levels at the end of the methanol fed-batch. In order to exclude cross regulations between sulfur and phosphorus metabolism or regulation resulting from a general stress reactions we additionally analyzed the influence of (NH_4_)_2_HPO_4_-supplementation on the transcript levels of sulfur limitation marker genes and vice versa. The transcript levels of phosphorus marker genes in the 1× and 2× SO_4_-supplemented cultures behaved like the reference. Only for *PHO84* the degree of induction seemed slightly lower, being 60-fold higher on average. The addition of (NH_4_)_2_HPO_4_ seemed to have a slight influence on the strenght of sulfur marker gene upregulation. At least for *JLP1*-*1* transcript levels were less strongly upregulated (20-fold vs. 35-fold in the reference culture), but overall the trends of regulation were unaffected (Fig. 9).

### Nitrogen supplementation is not responsible for improved growth and CpB production

Since we selected (NH_4_)_2_SO_4_ and (NH_4_)_2_HPO_4_ for increasing the sulfur or phosphorus-availability in the culture, an additional nitrogen source became available. We used these ammonium-based salts on the one hand to avoid further addion of metal ions and on the other hand to be able to analyze the transcriptional response of the identified potential nitrogen limitation marker genes upon nitrogen supplementation simultaneously. The given amounts of added nitrogen are referred to the total amount which has been added during the whole cultivation together with the respective SO_4_ or PO_4_ pulses [Reference: no additional nitrogen; 1× (NH_4_)_2_SO_4_: 20 mM; 2× (NH_4_)_2_SO_4_: 40 mM; 1× (NH_4_)_2_HPO_4_: 28 mM]. The nitrogen amount added in frame of the pH-control during the cultivation was comparable for the reference and SO_4_-supplemented cultures (33 mL ± 4 mL of 25% NH_3_ which is equal to 364 ± 44 mM). Only the PO_4_-supplemented cultures received markedly less (21 mL; equal to 231 mM) during the cultivation. This calculation verifies that nitrogen addition in the course of (NH_4_)_2_SO_4_ and (NH_4_)_2_HPO_4_ supplementation can be neglected and that nitrogen is not the driving force for improved CpB production. The highest CpB levels were observed in (NH_4_)_2_HPO_4_-supplemented cultures which received about 40% less nitrogen than (NH_4_)_2_SO_4_-supplemented cultures.

In the reference culture e.g. transcript levels of polyamine transporter *TPO2* was 45-fold higher than in the glycerol fed-batch phase and was reduced to about tenfold higher levels in the (NH_4_)_2_HPO_4_-supplemented culture. Also proline oxidase *PUT1* levels decreased from 25× to less than 7× in all cultures with nitrogen supplementation. The transcript levels of *DUR3*-*2* and *DAL1* were not influenced. However, the neglectable nitrogen addition makes evaluation of marker gene regulation in respect of added nitrogen in frame of (NH_4_)_2_SO_4_ and (NH_4_)_2_HPO_4_ supplementation not possible.

## Discussion

### Upregulated high affinity transport systems and induction of alternative nutrient source utilization revealed limitations of phosphorus-, sulfur- and nitrogen during fed-batch cultivations of recombinant *P. pastoris*

Sensing of culture conditions and adaptations of cellular metabolism to nutrient availability have been extensively studied in *S. cerevisiae* and have revealed that many cellular processes such as nutrient utilization, ribosome biogenesis, and stress responses are directly affected. As a result, cell cycle progression is modified [37]. In consequence, limitations in macronutrients or even starvation conditions might affect cellular growth and recombinant protein production (RPP) in yeast production platforms. To detect and overcome such limitations, different approaches for media optimization of complex or synthetic media for use in an industrially relevant setup have been experimentally tested. They were mainly based on host physiology and the fermentation performance [11]. Important considerations were the cellular composition of the host [38] but the impact of recombinant protein production in a relevant production process had not been considered. In the present work transcriptomics studies of a production process of the recombinant enzyme CpB in carbon-limited fed-batch cultivations of *P. pastoris* showed that methanol- and glucose-grown cultures experience transcriptional upregulation of genes connected to phosphorus and nitrogen metabolism from the middle to the end of the fed-batch. Unique for the methanol fed-batch was a strong upregulation of sulfur associated genes. The strong upregulation of the respective genes was not only clearly observed during the later methanol fed-batch, but also during the methanol induction phase. The mentioned genes are connected to transport processes and utilization of alternative substrates for coverage of the phosphorus- nitrogen-, and sulfur demand. Whereas gene regulation of phosphorus associated genes were similar between methanol and glucose fed-batch, upregulation of nitrogen associated genes was stronger on methanol and upregulation of sulfur-associated genes was only visible in the methanol fed-batch. Tracking those indications, principle component analysis (PCA) revealed connections to permanent limitations on macronutrient level which occur at high cell densities when nutrients are used up in the cellular environment which leads to upregulation of alternative pathways for coverage of respective demands.

The IAM medium was designed based on cellular composition of *P. pastoris* [26] and has successfully been used for production of several model proteins in *P. pastoris* [8, 19, 39]. Considerations for media development were a balanced composition based on the elemental composition of *P. pastoris*, comparably low, but sufficient salt concentration for growth, and suitability for downstream processing. Especially salt content and osmolarity can play an important role [21] as high salt concentration can inhibit growth [22] and herewith might also influence recombinant protein production. Furthermore, high salt concentrations make downstream processing more difficult [4]. Even proteolytic activity and consequently product stability in the supernatant can be influenced. By specific nutrient addition we intended to improve cellular growth and recombinant protein production by reduction of starvation-induced cellular changes which could reduce recombinant protein production on different levels [40, 41].

### Growth on methanol has higher and different nutrient demand than glucose metabolization

As metabolization of methanol is quite different to glucose consumption we were interested if such differences would result in a differing transcriptional response of selected marker genes. The observations indicate marked differences in the demand of nutrients even when developing equal biomass and using the same *P. pastoris* strain. This increased demand became even more obvious when analyzing the transcriptional response during the methanol induction phase where an increasing demand of respective nutrients was observed. When *P. pastoris* methanol metabolism is induced and the cells grow on methanol as sole carbon and energy source many cellular adaptations take place. They range from equipping the cell with the enzymatic apparatus for methanol metabolization [42] to organelle reconstruction (e.g. peroxisomes, vacuole, mitochondria) [36] which could lead to this increased nutrient and amino acid demand. These adaptations are also reflected by the higher protein content of methanol grown cells [33]. Furthermore there is a higher demand of cofactors, needed for growth on methanol. Among them, glutathione as redox regulator, representing a major cellular sulfur containing compound, is essential for prevention of cell damage. As glutathione levels are elevated in *P. pastoris* grown on methanol [43], compared to glucose, this could explain the high sulfur demand. Some cellular or metabolic changes during the adaptations to methanol might be only temporary and can be compensated by upregulation of transport processes to a certain point. However, the difference in the strength of upregulation between methanol and glucose grown cells in the later fed-batch can be caused by the early consumption of available nutrients during the methanol induction phase which makes the transcriptional response earlier and stronger. This implies that an appropriate media supplementation, especially for high producing strains like a P_*AOX1*_-based expression system, could have an even stronger positive impact on growth and recombinant protein production compared to a P_GAP_-based system. The higher load of recombinant protein synthesis and induced stress reactions could result in an even higher demand for the mentioned media components.

### Phosphorus

Inorganic phosphate (Pi) represents an important macronutrient for cellular metabolism in different purposes. Basically it is utilized for energy metabolism in terms of ATP generation but is also needed as cofactor for enzymes, nucleoproteins, or as intracellular buffer [44]. Phosphate homeostasis in *S. cerevisiae* can be divided into (1) transport processes of Pi or alternative substrates through the plasma membrane, (2) adaptation of the metabolism on transcriptional level by the PHO regulon, and (3) vacuolar release of inorganic phosphate. Uptake of Pi by yeast cells is mediated by a set of phosphate-specific transporters or cotransporters. The major storage organelle of excess phosphorus is the vacuole. Under limiting Pi levels, the PHO pathway becomes activated by Pho4 together with Pho2. This leads to increased expression of genes associated with acquisition, uptake and storage of Pi. The activity of Pho4 is regulated by the Pho80–Pho85 cyclin dependent kinase complex (CDK). In consequence, high affinity transporters (Pho84 and Pho89) are activated and low affinity transporters (Pho87 and Pho90) inactivated. Low affinity transporters are targeted to the vacuole and become degraded. Furthermore the VTC-complex is induced which leads to increased polyphosphate (polyP) synthesis in the vacuole. For usage of alternative Pi sources for growth, Gde1 hydrolyzes glycerophosphocholine into choline and glycerophosphate [44].

The described effects of high- (glycerol fed-batch phase) or low Pi-conditions (late methanol fed-batch) are reflected by the transcriptional regulation of potential phosphorus limitation marker genes observed in our study. Strong upregulation was seen for the genes encoding the high affinity phosphate transporters *PHO84* and *PHO89*, a subunit of the VTC complex (*VTC1*) for release of Pi from the vacuole and *GDE1* involved in generation of Pi from alternative substrates. As phosphorus is an essential nutrient, the impact of limitations on cellular growth have been investigated. In *S. cerevisiae* phosphorus limitation lead to a decreased phosphorus content in the cell, the cell wall composition was lower in glucans but with increased protein content, changes in lipid compositions occured [45], and even cell size changed [46]. Furthermore it is known that growth continues to a certain amount, dependent on the previous amount of available phosphorus in the medium [47], but permanent restriction can result in growth arrest [46] resulting in downregulation of many processes that might also be rate limiting for recombinant protein production and secretion.

### Sulfur

Yeasts utilize sulfur for generation of sulfur amino acids and glutathione, which is acting as scavenger for oxygen free radicals. Metabolization occurs in frame of the assimilatory reduction from inorganic sulfur sources (e.g. sulfate) or organic sulfur sources (e.g. methionine). Uptake of sulfate is mediated by sulfate transport systems. Sulfate becomes reduced and then gets incorporated into organic compounds. As sulfur represents an essential nutrient, limitations in sulfur initially lead to upregulated transport into the cell, and in case of depletion of the preferred source of sulfur (inorganic, here sulfate) to uptake of alternative sulfur sources (organic sources). The uptake of sulfate is mediated, in dependency on its availability, by a high affinity and a low-affinity uptake system. In conditions with sulfur excess, glutathione can act as storage form and can be used under sulfur limitation for biosynthesis of sulfur containing amino acids [48, 49].

In this study, an increased sulfur demand on transcriptional level was observed at the beginning and in the later methanol fed-batch, but not during the glucose fed-batch. Genes with respective regulation were *SUL1, JLP1*-*1*, *MUP1*-*1*, *MUP3, MET4*, *MET32*, and *YCT1*. Sul1 represents a high affinity sulfate permease which controls the concentration of endogenous activated sulfate intermediates [50, 51] and is highly induced upon sulfate starvation in *S. cerevisiae* [31]. Jlp1 is a sulfonate/alpha-ketoglutarate dioxygenase involved in sulfonate catabolism for use as a sulfur source which is induced by sulfur starvation [52, 53]. Mup1 represents a high affinity methionine permease which is also involved in cysteine uptake [54] and was highly upregulated under sulfur starvation in *S. cerevisiae* [31]. Transcriptional regulation of MET-genes as occurring 1 h after the start of the methanol-feed was already described in correlation to methanol metabolism in *P. pastoris* [55]. The regulatory pattern observed towards the end of the fed-batch is not necessarily connected to the methanol metabolism as Met4 is the only transcriptional activator of sulfur metabolism [56] and upregulation of *MET4* and *MET32,* a transcription factor involved in transcriptional regulation of the methionine biosynthetic pathway was described under sulfur starvation in *S. cerevisiae* [31]. Similar gene regulations were also observed in *Yarrowia lipolytica* [57]. Based on these results we selected *JLP1*-*1*, *SUL1*, *MUP1*-*1* and *MET32* as potential limitation markers in fed-batch cultivations. Respective supplementation led to strongly reduced transcript levels of all selected marker genes at the end of the fed batch, which were dose-dependent (1×, 2× sulfur supplementation), and also had a positive impact on biomass generation and CpB levels in culture supernatants. A connection between sulfur limitation and the impact on biomass formation is given since sulfur metabolic flux is correlated to growth rate and it can have impact on initiation of cell division in *S. cerevisiae* [58]. In *Trichoderma reesei* it was even proposed that during recombinant protein production, which requires increased methionine and cysteine levels, sulfur assimilation could be limiting [59].

### Nitrogen

Microbial cellular compounds containing nitrogen are mainly amino acids, purines and pyrimidines. Yeasts are able to utilize different nitrogen sources to cover their demand. In *S. cerevisiae* nitrogen can be taken up e.g. in form of urea and ammonium or as amino acids (proline or arginine). The quality of the nitrogen source further determines the maximum growth rate and has profound impact on cellular growth. For the biosynthesis of nitrogenous compounds, the potential nitrogen sources are further metabolized to glutamate or glutamine [60]. Contrary to other methylotrophic yeasts, *P. pastoris* cannot assimilate nitrate [61], and is also deficient in utilization of urea.

Depending on the availability of a preferred nitrogen source (excess or limitation) or a non-preferred nitrogen source, respective pathways for homeostasis of nitrogen are regulated. Upon limitation, upregulated cellular functions are associated with the vacuole, cell cycle, and organelle organization/biogenesis. The transcriptional response of a nitrogen-limited culture is similar to the nitrogen catabolite repression and results in upregulation of several pathways. These pathways are the allantoin pathway (DAL genes), proline utilization genes (*PUT1/2*), glutamate metabolizing genes (*GLT1*/*GDH1*), and genes for amino acid and ammonium transporters (as *GAP1*, *MEP2*, *VBA1*, *AVT1*/*4*) [62]. In our dataset we additionally observed a marked upregulation of *TPO2*, a polyamine transporter, responsible for the uptake of polyamines as alternative substrate for covering the nitrogen demand. Supplementation with different amounts of nitrogen in form of (NH_4_)_2_HPO_4_ or (NH_4_)_2_SO_4_ led to decreased transcript levels of *TPO2*, *PUT1* but did not affect *DUR3*-*1*, and *DAL1*. In the described and analyzed processes, additional nitrogen supply in the course of sulfur and phosphorus supplementation did not play a major role but should nevertheless be considered as crucial as it has been reported that nitrogen availability and the type of nitrogen source has an effect on gene expression strength of MUT and PEX genes in *P. pastoris* [63]. Furthermore nitrogen starvation was found to affect ribosomal protein production [62], their degradation [23] and to slow down translation rates [64]. Also, degradation of already secreted proteins could become an issue since expression and secretion of proteases takes place in nitrogen limited conditions [65] which could also affect product stability in culture supernatants [66].

## Conclusions

Transcriptomics analyses have shown that sulfur-, phosphorus- and nitrogen limitation occur in common methanol- or glucose-limited fed-batch processes of recombinant *P. pastoris*, being more pronounced in the methanol fed-batch. We identified biomarkers for these nutrient limitations, that can be versatily used for monitoring potential deficiencies occurring during production processes. Using qPCR of these marker genes as novel method for detection of nutrient limitations we were able to rectify such limitations on transcriptional level by addition of (NH_4_)_2_HPO_4_ or (NH_4_)_2_SO_4_. Sulfur and phosphorus supplementation but not nitrogen addition led to improved cellular growth and recombinant protein production. This approach is not limited to processes for recombinant protein production, but can be also used for other strains or production hosts as the need for tailor-made cultivation media is emerging. Applicability, especially for high producing cell lines, is conceivable as stress levels increase the demand for optimal culture conditions. In this regards, an appropriate supplementation can lead to improved recombinant protein production by diminishing the higher metabolic burden due to the strong production of recombinant protein.

## Methods

### Strain

*Pichia pastoris* (*Komagataella phaffii*) CBS7435 Mut^+^ (methanol utilization plus phenotype), producing porcine pro-carboxypeptidase B (CpB) as model protein under the control of the P_GAP_ promoter and the *S. cerevisiae* alpha mating factor as secretion signal, was selected for the described bioreactor cultivations.

### Fed batch cultivations

The preculture (100 mL YPG + 50 µg mL^−1^ Zeocin) was inoculated using a 1 mL cryo stock and was cultivated overnight at 150 rpm. The exponentially growing culture was washed (glycerol batch medium) and used for inoculation of the bioreactor culture to OD 1. Bioreactor cultivations were performed in a 2.7 L DASGIP bioreactor with a batch volume of 1.225 L for the inital cultivations used for transcriptomics analyses, or in 1 L bioreactors with a batch volume of 0.325 L (DASGIP Parallel Bioreactor System, Germany) for the other cultivations. The fed-batch process comprised four phases, starting with a glycerol-batch phase. The initial cultivations for microarray analyses were performed using the following setup:

After reaching the batch end at about 24 h, a carbon limited glycerol fed-batch for 5 h was started with a feed rate of 5.5 g L^−1^ h^−1^ to reach a biomass of 40 g L^−1^ dry cell weight (DCW). Methanol-grown cultures were then supplemented with 0.5% (w/v) methanol for induction of methanol metabolism. After consumption of the methanol shot, a methanol fed-batch was started and cultures were grown for 67 h to reach a final biomass of 100 g L^−1^. The glucose fed-batch was started with an initial feed rate of 1.9 g L^−1^ h^−1^ for 4 h to generate the same biomass as the methanol shotted cultures. The feed rate of the methanol- or glucose fed-batch medium during the fed-batch was increased after 14 h (methanol FB: initial feed rate 6.8 g L^−1^ h^−1^, feed increase after 12 h to 9.0 g L^−1^ h^−1^; glucose FB: initial feed rate 9.9 g L^−1^ h^−1^, feed increase after 14 h to 13.2 g L^−1^ h^−1^). Due to insolubility of salts (see salt shots and supplements) in the methanol fed-batch medium, methanol and glucose grown cultures were supplemented with respective amounts representing a surplus for generation of about 10 g yeast biomass (DCW).

The initial cultivations were performed in triplicates at 25 °C. Dissolved oxygen was controlled at 20%, and pH was adjusted to 5 by 25% NH_3_. Foam formation was prevented by addition of Glanapon 2000 (5%; Bussetti & Co GmbH, Austria) upon demand using a level probe. Samples were taken at the batch end, at the end of the glycerol fed-batch, and 1, 15, 27 41, 53 and 67 h after starting the methanol- and glucose fed-batch.

The cultivations for media supplementation were performed in duplicates and were carried out at the same setpoints. The fed-batch process was performed identically to the initial cultivations, only the feed-rate during the fed-batches were adapted to the smaller scale. During the methanol fed-batch, the cultures were supplemented with a sulfur and/or phosphorus source after each sampling.

### Cultivation media and supplements

Preculture medium contained per liter: 20 g soy peptone and 10 g yeast extract. Sterile glycerol stock was added after heat sterilization in autoclave (121 °C, 20 min) for a final glycerol concentration of 1.26%. Zeocin was used as selective agent (50 µg mL^−1^).

Glycerol batch medium (IAM) contained per liter: 2 g citric acid monohydrate, 45.6 g glycerol (86%), 12.6 g (NH4)_2_HPO_4_, 0.5 g MgSO_4_∙7H_2_O, 0.9 g KCl, 0.022 g CaCl_2_∙2H_2_O, 2 mL biotin (0.2 g L^−1^) and 4.6 mL PTM trace salts stock solution. HCl was used to set the pH to 5.0.

Glycerol fed-batch medium (GLY01) contained per liter: 724.4 g glycerol (86%), 12 mL PTM and 2 mL biotin (0.2 g L^−1^).

Methanol fed-batch medium (MET01) contained per liter: 835.1 mL pure methanol, 12 mL PTM, and 2 mL biotin (0.2 g L^−1^).

Glucose fed-batch medium (GLU04) contained per liter: 464.4 g L^−1^ glucose monohydrate, 12 mL PTM, and 2 mL biotin (0.2 g L^−1^).

PTM trace salts stock solution contained per liter: 6.0 g CuSO_4_·5H_2_O, 0.08 g NaI, 3.0 g MnSO_4_·H_2_O, 0.2 g Na_2_MoO_4_·2H_2_O, 0.02 g H_3_BO_3_, 0.5 g CoCl_2_, 20.0 g ZnCl_2_, 5.0 g FeSO_4_·7H_2_O, and 5.0 mL H_2_SO_4_ (95–98% w/w).

Salt shots contained per liter: 13.9 g MgSO_4_·7H_2_O, 27.7 g KCl and 0.69 g CaCl_2_∙2H_2_O. For solubility reasons in methanol fed-batch medium, respective salts were provided in shots (22.5 mL) for the reference culture or got combined with respective supplements for the sulfur and phosphorus-supplemented cultures.

Sulfur supplement and common salt shot were prepared as one solution which contained per liter: 91.5 g (NH_4_)_2_SO_4_, 13.9 g MgSO_4_·7H_2_O, 27.7 g KCl, and 0.69 g CaCl_2_·2H_2_O. Sulfur supplements were pulsed at 6 time points (22.5 mL) during the fed-batch process directly after a sampling.

Phosphorus supplement was prepared and added separately from common salt shot due to insolubility of (NH_4_)_2_HPO_4_ in the common salt shot. Phosphorus supplement contained per liter: 392 g (NH_4_)_2_HPO_4_. The common salt shot contained per liter: 20.8 g MgSO_4_·7H_2_O, 41.6 g KCl, and 1.04 g CaCl·2H_2_O. Phosphorus supplements were pulsed at 6 time points (7.5 mL) together with 15 mL common salt shot during the fed-batch process directly after a sampling.

### Biomass determination

Dry cell weight (DCW) was determined in triplicates by sampling of 2 mL cell suspension into pre-weighed and pre-incubated (1 d, 100 °C) reaction tubes and subsequent washing of cell pellet. DCW was determined after 3 days of drying at 100 °C.

### Enzymatic CpB assay

Quantification of recombinant product in culture supernatants was done using an enzymatic assay detecting CpB-activity. Prior to activity determination, supernatants were desalted using Zeba™ Spin columns (ThermoFisher Scientific, 2 mL, 7K MWCO) and pro-CpB was activated by trypsin (30 min, 37 °C). Due to the very sensitive assay principle, reactions were performed in cuvettes and CpB-activity was measured by the spectrophotometric method of Folk et al. [67] where the reaction velocity is determined by an increase in absorbance at 254 nm resulting from the hydrolysis of hippuryl-l-arginine. One unit CpB causes the hydrolysis of 1 µmol of hippuryl-l-arginine per minute at 25 °C and pH 7.65. All samples were measured in triplicates and activity was correlated to product concentrations using a CpB standard ranging from 0 to 69 mg L^−1^.

### SDS-PAGE

Qualitative analysis of culture supernatants was performed by sodium dodecyl sulfate–polyacrylamide gel electrophoresis (SDS-PAGE) using the NuPAGE Bis–Tris system (MOPS buffer). Samples were heat denatured (10′, 95 °C) and reduced. A BenchMark™ Protein Ladder (Thermo Fisher Scientific) was used. Proteins were separated (180 V, 70 min) and stained using PageBlue™ Protein Staining Solution (Thermo Fisher Scientific). After electrophoresis the gel was washed 3 times with deionized water, followed by staining with Coomassie brilliant blue and destained with deionized water.

### Viability measurements

Culture viability was determined using a flow cytometry-based method (BD™ Cell Viability kit). Bioreactor samples were stored on ice and cells were then separated by ultrasonication (3 × 6 s, 85% amplitude) and stained with propidium iodide. Flow cytometric analysis was performed at a Gallios™ cytometer (Beckman Coulter).

### Microarray experiments

Total RNA was isolated from phenol/ethanol-fixated cells using TRI reagent according to the supplier’s instructions (Ambion, USA). Isolated RNA was quantified by Nanodrop and integrity determined by Bioanalyzer (Agilent) or gel electrophoresis. For analysis of gene regulation with DNA-microarray, RNA was labelled with the fluorescent dyes Cy3 and Cy5, transcribed to cDNA, then to cRNA and finally purified (according to Two-Color Microarray-Based Gene Expression Analysis manual, Agilent). After a final quality control (integrity and labelling efficiency) cRNAs were hybridized to *P. pastoris*-specific microarray glass slides (8x15k-array, AMAD-ID: G2509F, Agilent), together with a reference pool-cRNA sample, and fluorescence intensity was measured via microarray scanner (Agilent). Raw data were normalized using variance stabilising normalisation (VSN) using the R-platform and then integrated into the *P. pastoris* database [68].

Normalization steps and statistical analysis of microarray data included removal of color bias using locally weighted MA-scatterplot smoothing (LOESS) followed by between array normalization using the “Aquantile” method. The P values associated with the differential expression values were calculated using a linear model fit (limma R package), subsequently they were adjusted for multiple testing using the method of Benjamini and Yekutieli [69] using the BY method of limma R package. To identify differentially expressed genes, the following criteria were applied: fold change cut-off of at least 1.5  >  FC > 1/1.5 and adjusted P value <0.05. All steps were performed using the R-software package [70], and the limma package.

### Principle component analysis (PCA)

Principle component analysis was performed on the microarray dataset present in log_2_FC referred to the glycerol fed-batch phase using the Microsoft Excel plugin XLSTAT.

### Quantitative real-time PCR

Quantitative RT-PCR was used for transcript level determination of the reference cultivations and supplemented cultures. RNA from phenol/ethanol fixed samples was isolated, quantified and integrity was checked by agarose gel electrophoresis. After DNase treatment, RNA was transcribed to cDNA using the SensiMix SYBR Kit (QT605-05, Bioline, UK). Analysis was performed at a Rotor Gene 6000 real-time PCR cycler (Qiagen, DE). All samples were analyzed in tri- or quadruplicates.

The used protocol includes the following steps:

1. Hold (95 °C; 10 min), 2. Cycling (45 repeats including denaturation: 95 °C; 15 s, primer annealing: 60 °C; 20 s, and elongation: 72 °C; 15 s), and 3. Melting (65 °C; 90 s; Ramp from 65 to 99 °C incrementally increased by 0.5 °C (each 2 s).

The occurrence of primer dimers and the purity of the PCR product were checked by melting curve analysis and raw data were normalized against *ACT1* as housekeeping gene and referenced to the sample of the glycerol fed-batch (according to the double delta Ct method for relative quantification [71]). Primer sequences can be found in the Additional file 1: Table S3.

## Additional file

**Additional file 1: Figure S1.** Mean expression values (microarray data) of the carboxypeptidase B gene during the methanol and glucose fed-batch process. **Table S2.** Description of biological function of marker genes adapted from Saccharomyces Genome Database (SGD). **Table S3.** qPCR primer sequences. **Figure S4.** Principal component analysis (variable factor map; F2 vs. F1) of the methanol fed-batch (1 h, 15 h, 27 h, 41 h, 53 h and 67 h after starting the methanol fed-batch) referred to the glycerol fed-batch (log_2_ fold change). **Figure S5.** Principal component analysis (variable factor map; F3 vs. F2) of the methanol fed-batch (1 h, 15 h, 27 h, 41 h, 53 h and 67 h after starting the methanol fed-batch) referred to the glycerol fed-batch (log_2_ fold change). **Figure S6.** Principal component analysis (variable factor map; F2 vs. F1) of the glucose fed-batch (1 h, 15 h, 27 h, 41 h, 53 h and 67 h after starting the glucose fed-batch) referred to the glycerol fed-batch (log_2_ fold change). **Figure S7.** Principal component analysis (variable factor map; F3 vs. F2) of the glucose fed-batch (1 h, 15 h, 27 h, 41 h, 53 h and 67 h after starting the glucose fed-batch) referred to the glycerol fed-batch (log_2_ fold change).

## Abbreviations

RPP: recombinant protein production
CpB: pro-carboxypeptidase B
BSM: basal salt medium
DOE: design of experiments
PCA: principle component analysis
DCW: dry cell-weight
Pi: inorganic phosphate
VSN: variance stabilizing normalization
