# Supplementary material for: Biomarkers allow detection of nutrient limitations and respective supplementation for elimination in Pichia pastoris fed-batch cultures
Source: Microb Cell Fact. 2017 Jul 11;16:117. doi: 10.1186/s12934-017-0730-9 (PMC5504661; doi:10.1186/s12934-017-0730-9)
Supplement: Supplementary file 1 — Additional file 1: Figure S1. Mean expression values (microarray data) of the carboxypeptidase B gene during the methanol and glucose fed-batch process. Table S2. Description of biological function of marker genes adapted from Saccharomyces Genome Database (SGD). Table S3. qPCR primer sequences. Figure S4. Principal component analysis (variable factor map; F2 vs. F1) of the methanol fed-batch (1 h, 15 h, 27 h, 41 h, 53 h and 67 h after starting the methanol fed-batch) referred to the glycerol fed-batch (log2 fold change). Figure S5. Principal component analysis (variable factor map; F3 vs. F2) of the methanol fed-batch (1 h, 15 h, 27 h, 41 h, 53 h and 67 h after starting the methanol fed-batch) referred to the glycerol fed-batch (log2 fold change). Figure S6. Principal component analysis (variable factor map; F2 vs. F1) of the glucose fed-batch (1 h, 15 h, 27 h, 41 h, 53 h and 67 h after starting the glucose fed-batch) referred to the glycerol fed-batch (log2 fold change). Figure S7. Principal component analysis (variable factor map; F3 vs. F2) of the glucose fed-batch (1 h, 15 h, 27 h, 41 h, 53 h and 67 h after starting the glucose fed-batch) referred to the glycerol fed-batch (log2 fold change). [file 12934_2017_730_MOESM1_ESM.pdf]

Biomarkers allow detection of nutrient limitations and respective supplementation for elimination in *Pichia pastoris* fed-batch cultures

Jonas Burgard, Minoska Valli, Alexandra B. Graf, Brigitte Gasser, Diethard Mattanovich

Additional Figures and Tables

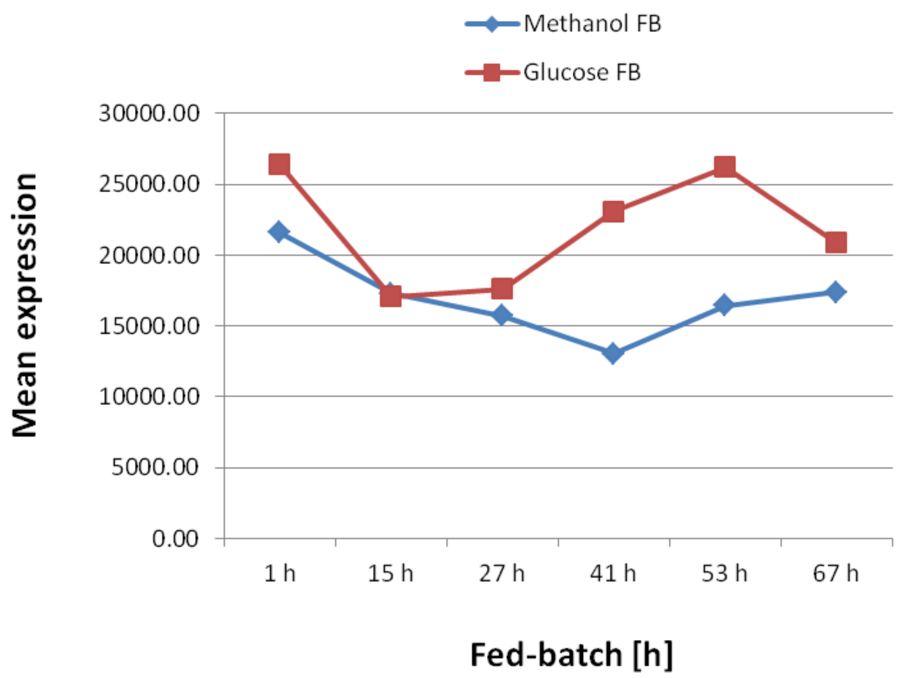

Additional Figure S1: Mean expression values (microarray data) of the carboxypeptidase B during the methanol and glucose fed-batch process

Additional Table S2: Description of biological function of marker genes adapted from *Saccharomyces* Genome Database (SGD)

ORF name	Description
**PHO84**	High-affinity inorganic phosphate (Pi) transporter; also low-affinity manganese transporter; regulated by Pho4p and Spt7p
**PHO89**	Plasma membrane Na+/Pi cotransporter; active in early growth phase; transcription regulated by inorganic phosphate concentrations and Pho4p
**VTC1**	Regulatory subunit of the vacuolar transporter chaperone (VTC) complex; VTC complex is involved in membrane trafficking, vacuolar polyphosphate accumulation, microautophagy and non-autophagic vacuolar fusion
**PHO80**	Cyclin; interacts with cyclin-dependent kinase Pho85p; regulates the response to nutrient levels and environmental conditions, including the response to phosphate limitation and stress-dependent calcium signaling
**PHO81**	Cyclin-dependent kinase (CDK) inhibitor; regulates Pho80p-Pho85p and Pcl7p-Pho85p cyclin-CDK complexes in response to phosphate levels
**GDE1**	Glycerophosphocholine (GroPCho) phosphodiesterase; hydrolyzes GroPCho to choline and glycerolphosphate, for use as a phosphate source and as a precursor for phosphocholine synthesis
**PHO5**	Repressible acid phosphatase; 1 of 3 repressible acid phosphatases that also mediates extracellular nucleotide-derived phosphate hydrolysis; induced by phosphate starvation and coordinately regulated by PHO4 and PHO2
**DAL4**	Allantoin permease; expression sensitive to nitrogen catabolite repression and induced by allophanate, an intermediate in allantoin degradation
**DAL5-2**	Allantoate permease; ureidosuccinate permease; also transports dipeptides, though with lower affinity than for allantoate and ureidosuccinate; expression is constitutive but sensitive to nitrogen catabolite repression
**DUR3-1**	Plasma membrane transporter for both urea and polyamines; expression is highly sensitive to nitrogen catabolite repression and induced by allophanate, the last intermediate of the allantoin degradative pathway
**DUR3-2**	Plasma membrane transporter for both urea and polyamines, expression is highly sensitive to nitrogen catabolite repression and induced by allophanate, the last

	intermediate of the allantoin degradative pathway
**TPO2**	Polyamine transporter of the major facilitator superfamily; specific for spermine; localizes to the plasma membrane
**PUT1**	Proline oxidase; nuclear-encoded mitochondrial protein involved in utilization of proline as sole nitrogen source; PUT1 transcription is induced by Put3p in the presence of proline and the absence of a preferred nitrogen source
**CAR1-1**	Arginase, catabolizes arginine to ornithine and urea; expression responds to both induction by arginine and nitrogen catabolite repression
**GLT1**	NAD(+)-dependent glutamate synthase (GOGAT); synthesizes glutamate from glutamine and alpha-ketoglutarate; with Gln1p, forms the secondary pathway for glutamate biosynthesis from ammonia; expression regulated by nitrogen source; assembles into filaments as cells approach stationary phase and under cytosolic acidification and starvation conditions
**DAL1**	Allantoinase; converts allantoin to allantoate in the first step of allantoin degradation; expression sensitive to nitrogen catabolite repression
**SEO1-1**	Putative permease, member of the allantoate transporter subfamily of the major facilitator superfamily
**GAT1**	Transcriptional activator of nitrogen catabolite repression genes; contains a GATA-1-type zinc finger DNA-binding motif; activity and localization regulated by nitrogen limitation and Ure2p
**YBR139W**	Putative serine type carboxypeptidase; role in phytochelatin synthesis; green fluorescent protein (GFP)-fusion protein localizes to the vacuole; expression induced by nitrogen limitation in a GLN3, GAT1-independent manner
**JLP1-1**	Fe(II)-dependent sulfonate/alpha-ketoglutarate dioxygenase; involved in sulfonate catabolism for use as a sulfur source; contains sequence that resembles a J domain (typified by the E. coli DnaJ protein); induced by sulphur starvation
**MET32**	Zinc-finger DNA-binding transcription factor; involved in transcriptional regulation of the methionine biosynthetic genes; targets strong transcriptional activator Met4p to promoters of sulfur metabolic genes; feedforward loop exists in the regulation of genes controlled by Met4p and Met32p
**MET4**	Leucine-zipper transcriptional activator; responsible for regulation of sulfur amino acid pathway; requires different combinations of auxiliary factors Cbf1p,

	Met28p, Met31p and Met32p
**SUL1**	High affinity sulfate permease of the SulP anion transporter family; sulfate uptake is mediated by specific sulfate transporters Sul1p and Sul2p, which control the concentration of endogenous activated sulfate intermediates
**MUP1-1**	High affinity methionine permease; integral membrane protein with 13 putative membrane-spanning regions; also involved in cysteine uptake
**MUP3**	Low affinity methionine permease; similar to Mup1p
**YCT1**	High-affinity cysteine-specific transporter; green fluorescent protein (GFP)-fusion protein localizes to the endoplasmic reticulum

Additional Table S3: qPCR primer sequences

Primer name	Primer sequence
ACT1_Fwd	CCTGAGGCTTTGTTCCACCCATCT
ACT1_Rev	GGAACATAGTAGTACCACCGGACATAACGA
JLP1-1_Fwd	AGCATGGGACAACAGAAG
JLP1-1_Rev	TCAGCAAGAGGGGTAATTCG
MET32_Fwd	TTCTCCGACATCCTCCAT
MET32_Rev	AAGCCCTTCTGACATCTG
SUL1_Fwd	GATTTTACCTCCGCCAACTG
SUL1_Rev	CTGTCTCTCCTCTTCTTGTGT
MUP1-1 Fwd	GTCATCAAGTCACCAATCGT
MUP1-1 Rev	AGGCAGCAACACAGTGAA
PHO84-1_Fwd	AACCTCCCCAATACCACAAC
PHO84-1_Rev	GGCTCCCACTTTACCAGAA
VTC1_Fwd	AATTTCCGCTGGTCTGT
VTC1_Rev	GGCCCAAAACGGTCAT
PHO81_Fwd	AGCTCTCGTCAAACTATTCAG
PHO81_Rev	CAAACACCACACCACTCA
PHO5_Fwd	TGCTTACCGAGAGATTATTGTG
PHO5_Rev	AGGGCCAGAGGAACAGTCA
DUR3-1 _Fwd	CAGCATCCTCCTTCCTTATAC
DUR3-1_Rev	AAGCAGCAACACTAAACCAA
TPO2_Fwd	CGGTCTTGCCTGTGGTATT
TPO2_Rev	ACCGAAGCTCCCCATATC

PUT1_Fwd	ATGGCAGACGATGTTACAC
PUT1_Rev	CCGTTCTCCTCCAATCTTCT
DAL1_Fwd	GGAATTGCATCGGTCGGTTT
DAL1_Rev	GGCAGTGTTCAGCGAAGT

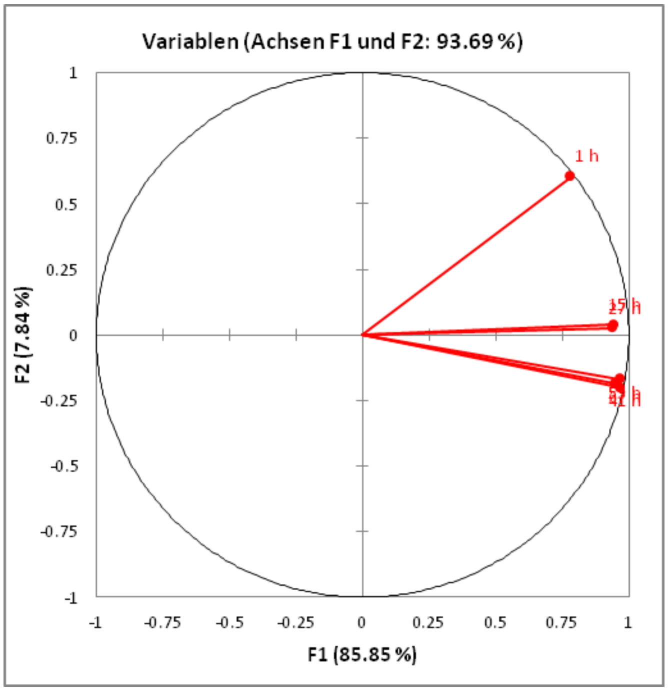

Additional Figure S4: Principal component analysis (variable factor map; F2 vs. F1) of the methanol fed-batch (1 h, 15 h, 27 h, 41 h, 53 h and 67 h after starting the methanol fed-batch) referred to the glycerol fed-batch (log2 fold change).

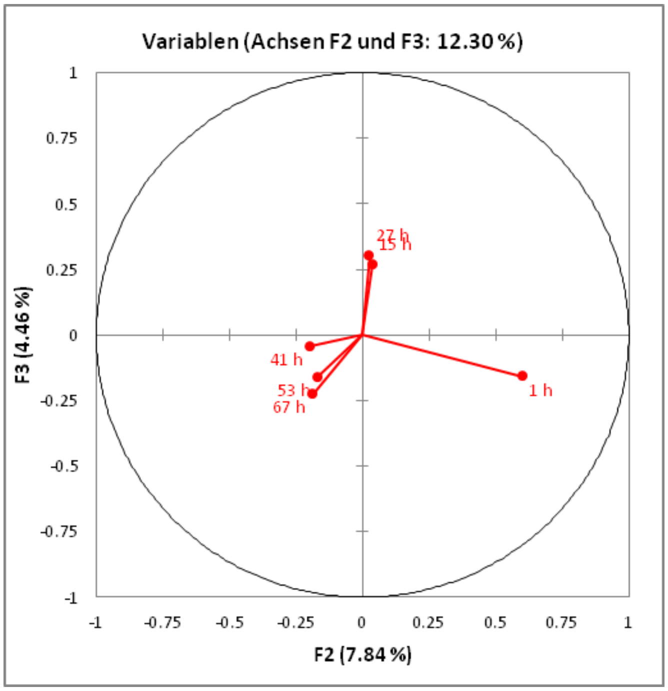

Additional Figure S5: Principal component analysis (variable factor map; F3 vs. F2) of the methanol fed-batch (1 h, 15 h, 27 h, 41 h, 53 h and 67 h after starting the methanol fed-batch) referred to the glycerol fed-batch (log2 fold change).

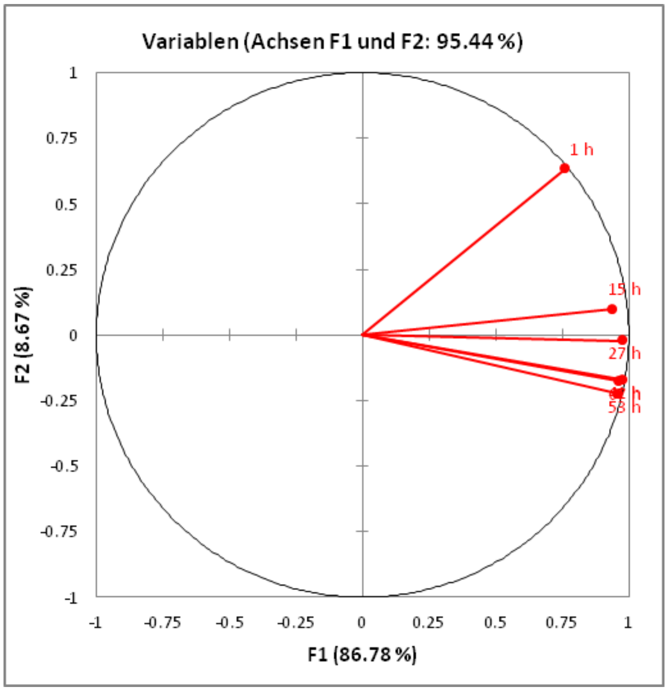

Additional Figure S6: Principal component analysis (variable factor map; F2 vs. F1) of the glucose fed-batch (1 h, 15 h, 27 h, 41 h, 53 h and 67 h after starting the glucose fed-batch) referred to the glycerol fed-batch (log2 fold change).

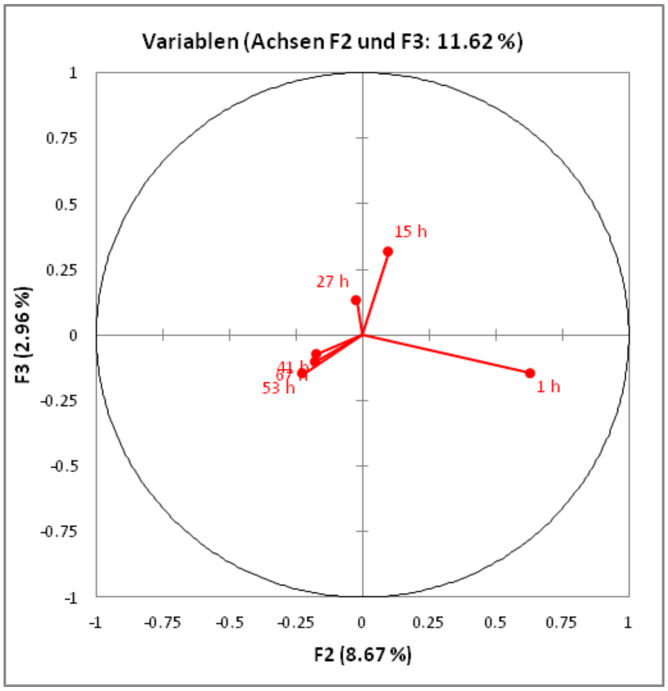

Additional Figure S7: Principal component analysis (variable factor map; F3 vs. F2) of the glucose fed-batch (1 h, 15 h, 27 h, 41 h, 53 h and 67 h after starting the glucose fed-batch) referred to the glycerol fed-batch (log2 fold change).
